# In vivo bioluminescence imaging of the spatial and temporal colonization of lactobacillus plantarum 423 and enterococcus mundtii ST4SA in the intestinal tract of mice

**DOI:** 10.1186/s12866-018-1315-4

**Published:** 2018-10-30

**Authors:** Winschau F. Van Zyl, Shelly M. Deane, Leon M. T. Dicks

**Affiliations:** 1 Department of Microbiology, Stellenbosch University, Private Bag X1, Matieland, Stellenbosch, 7600 South Africa; 2 Department of Microbiology, Stellenbosch University, Private Bag X1, 7 Matieland, Stellenbosch, 7602 South Africa

**Keywords:** Lactic acid bacteria, *Lactobacillus plantarum* 423, *Enterococcus mundtii* ST4SA, In vivo bioluminescence imaging, Luciferase, Gastrointestinal tract, Colonization, Intestinal persistence

## Abstract

**Background:**

Lactic acid bacteria (LAB) are major inhabitants and part of the normal microflora of the gastrointestinal tract (GIT) of humans and animals. Despite substantial evidence supporting the beneficial properties of LAB, only a few studies have addressed the migration and colonization of probiotic bacteria in the GIT. The reason for this is mostly due to the limitations, or lack of, efficient reporter systems. Here we describe the development and application of a non-invasive in vivo bioluminescence reporter system to study, in real-time, the spatial and temporal persistence of *Lactobacillus plantarum* 423 and *Enterococcus mundtii* ST4SA in the intestinal tract of mice.

**Results:**

This study reports on the application of the firefly luciferase gene (*ffluc*) from *Photinus pyralis* to develop luciferase-expressing *L. plantarum* 423 and *E. mundtii* ST4SA, using a *Lactococcus lactis* NICE system on a high copy number plasmid (pNZ8048) and strong constitutive lactate dehydrogenase gene promoters (P*ldh* and ST*ldh*). The reporter system was used for in vivo and ex vivo monitoring of both probiotic LAB strains in the GIT of mice after single and multiple oral administrations. *Enterococcus mundtii* ST4SA reached the large intestine 45 min after gavage, while *L. plantarum* 423 reached the cecum/colon after 90 min. Both strains predominantly colonized the cecum and colon after five consecutive daily administrations. *Enterococcus mundtii* ST4SA persisted in faeces at higher numbers and for more days compared to *L. plantarum* 423.

**Conclusions:**

Our findings demonstrate the efficiency of a high-copy number vector, constitutive promoters and bioluminescence imaging to study the colonization and persistence of *L. plantarum* 423 and *E. mundtii* ST4SA in the murine GIT. The system allowed us to differentiate between intestinal transit times of the two strains in the digestive tract. This is the first report of bioluminescence imaging of a luciferase-expressing *E. mundtii* strain to study colonization dynamics in the murine model. The bioluminescence system developed in this study may be used to study the in vivo colonization dynamics of other probiotic LAB.

**Electronic supplementary material:**

The online version of this article (10.1186/s12866-018-1315-4) contains supplementary material, which is available to authorized users.

## Background

Lactic acid bacteria are common inhabitants of a healthy human and animal gastrointestinal tract and they play a major role in keeping the gut microbiota in a balanced state [1–4]. Beneficial properties include the inhibition of enteric pathogens [5], alleviation of constipation [6] and diarrhoea [7], stimulation of the immune system [8], repression of cancer cell and tumor growth [9, 10], and synthesis of essential metabolites such as vitamins [11]. For probiotics to confer their beneficial effects on the host, they must be able to survive stomach acids and bile salts and persist at high levels in the intestinal tract [12]. Some strains have adapted to these harsh conditions by over-expressing specific genes when exposed to acids and bile salts [13–15].

The survival and colonization of LAB in the GIT is usually studied in vitro and ex vivo by using models simulating the GIT [16–19]. Although these studies are valuable in understanding the survival of LAB in the GIT, the findings seldom reflect real-life conditions. More in vivo studies are needed to understand the interactions between probiotic bacteria, pathogens, commensal bacteria and gut epithelial cells. The best approach to study real-time interactions between probiotic bacteria and their mammalian host in the GIT is by labelling the cells with fluorescent or bioluminescent markers [20–26]. A selection of genes, encoding proteins that emit light at specific wavelengths, are available for cloning into plasmids or insertion into the genomes of recipient cells [21, 22]. The most commonly used luciferase labelling systems used in in vivo and ex vivo tracking of bacteria are bacterial *luxABCDE* from *Photorabdus luminescence* [26], click beetle luciferase (CBluc) from *Pyrophorus plagiophthalamus* [23] and firefly luciferase (Ffluc) from *Photinus pyralis* [25]. The CBluc and Ffluc luciferases require the exogenous addition of D-Luciferin, whereas the *lux* substrate is synthesized by proteins encoded in the *lux* operon [20]. The half-life of luciferase is only several seconds and does not represent bioluminescence accumulated over a period [28]. Another advantage is that only low levels of background luminescence are emitted by mammalian tissue.

To date, very few studies have used either fluorescence or bioluminescence whole-body imaging to monitor the persistence of LAB in the GIT, and with variable degrees of success. This is mostly due to the weak penetration of photons through muscles and tissue. Furthermore, labelled cells orally administered are often dispersed throughout the GIT or become metabolically inactive and emit bioluminescent signals too weak to detect. Cronin et al. [29] used a bacterial *lux* system to study the persistence of *Bifidobacterium breve* in mice. The bioluminescent signal was, however, not emitted from the GIT of live mice and all imaging had to be done ex vivo after dissecting the GIT. In our own studies [24], fluorescence encoded by the *mCherry* gene, transformed into *Lactobacillus plantarum* 423 and *Enterococcus mundtii* ST4SA, was also only detected after surgical removal of the GIT. This is not unusual, as also reported by Oozeer et al. [26] and Corthier et al. [30] with studies done on mice. Lee and Moon [31] were one of the first to detect *Lc. lactis* in the GIT of live mice by using a pMG36e Ffluc plasmid vector, although the strain could only be detected for up to 2 h. Berlec and coworkers [25] successfully used the infrared fluorescent protein IRFP713 to monitor *Lactococcus lactis*, *L. plantarum* and *Escherichia coli* in the GIT of mice. The virulence gene expression and gut persistence abilities of two pathogenic *Enterococcus faecalis* strains were studied using the bacterial luxABCDE cassete [32].In another study, Daniel et al. [33] successfully monitored the colonization and persistence of *Lc. lactis* and *L. plantarum* in live mice using the CBluc luciferase system.

*Lactobacillus plantarum* 423, isolated from sorghum beer and *E. mundtii* ST4SA, isolated from soybeans both have probiotic properties [34–36]. The strains survive conditions in the human GIT, as shown with studies using a model simulating the intestinal conditions of infants [16]. Both strains adhere to human intestinal epithelial cells [37] and produce antimicrobial peptides [38, 39], active against *Listeria monocytogenes*, *Enterococcus faecalis*, *Clostridium sporogenes* and *Salmonella typhimurium* [34, 35, 40–42]. In a previous report, using the *mCherry* fluorescence gene, we have shown that *L. plantarum* 423 and *E. mundtii* ST4SA were localized in the cecum and colon of mice after a single oral dosage [24].

This study reports on the application of a red-shifted thermostable firefly luciferase-system (Ffluc) to study the spatial and temporal persistence of *L. plantarum* 423 and *E. mundtii* ST4SA in the GIT of mice after single and multiple dosages. The use of a red-emitting luciferase with a longer wavelength (620 nm) enabled optimal light penetration through intestinal and skin tissue. The in vitro and in vivo expression of the Ffluc system was optimized using a combination of a high-copy number plasmid vector and strong constitutive promoters. Differences between the two strains in viability and persistence in the GIT of mice were demonstrated by monitoring in vivo and ex vivo bioluminescence, using the Caliper in vivo imaging system (IVIS; Caliper Life Sciences, Hopkinton, MA). The bioluminescence system also allowed tracking of each of the strains in different sections of the GIT.

## Results

### In vitro functionality and stability of bioluminescent *L. plantarum* 423 and *E. mundtii* ST4SA

**Fig. 1:**
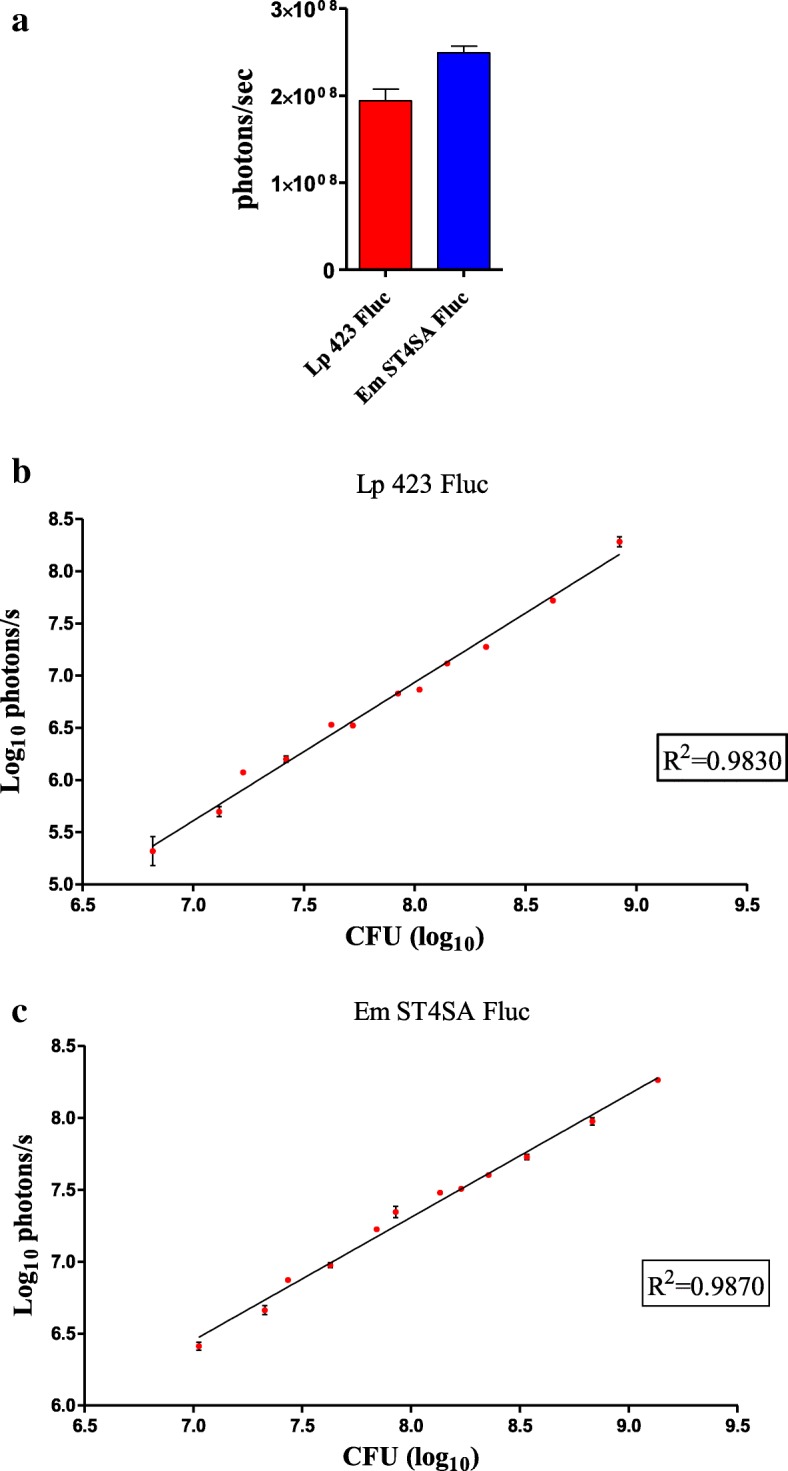
Quantification of bioluminescent *L. plantarum* 423 Fluc and *E. mundtii* ST4SA Fluc. **a** Bioluminescence measured in cultures of *L. plantarum* 423 Fluc and *E. mundtii* ST4SA Fluc distributed in black 96-well microtitre plates. Means from six independent cultures (4 × 10^9^ CFU per culture) are shown with standard deviations, and the background from each strain has been subtracted from each respective measurement. Correlation between bioluminescent signals and bacterial cell numbers of **(b)** *L. plantarum* 423 Fluc and **(c)** *E. mundtii* ST4SA Fluc. Cultures of each strain were serially diluted in black microplates and the bioluminescent signals quantified using the IVIS and then correlated with CFUs. Log_10_ averages of three cultures are plotted, with error bars indicating standard deviations. The logarithmic trendline and the correlation of determination (R^2^) between bioluminescence measurements and bacterial numbers of each strain are shown

Bacterial cultures resuspended in phosphate buffered saline (PBS) were used to image the intensity of bioluminescent signals produced by *L. plantarum* 423 Fluc and *E. mundtii* ST4SA Fluc (Fig. 1a). No significant difference was observed in the maximum intensities of bioluminescent signals produced by *L. plantarum* 423 Fluc or *E. mundtii* ST4SA Fluc. Maximum bioluminescence was recorded for *E. mundtii* ST4SA Fluc with a mean value of 2.49 × 10^8^ photons per second (p/s), while a slightly lower mean value of 1.94 × 10^8^ p/s was recorded for *L. plantarum* 423 Fluc. None of the control strains (*L. plantarum* 423 (pNZ8048) or *E. mundtii* ST4SA (pNZ8048)), emitted bioluminescent signals. No bioluminescence was detected in culture supernatants of *L. plantarum* 423 Fluc or *E. mundtii* ST4SA Fluc, indicating that bioluminescent light production was strictly intracellular.

Bioluminescence emitted by cells that expressed firefly luciferase correlated with serial dilutions of total CFUs of cultures of *L. plantarum* 423 Fluc (R^2^ = 0.9830) and *E. mundtii* ST4SA Fluc (R^2^ = 0.9870), indicating that photon emission accurately reflects bacterial cell numbers **(**Fig. 1b and c). The bioluminescence signal corresponded to the detection of bacterial CFU over a broad range, from approximately 6 × 10^6^ CFU to 8.5 × 10^8^ CFU for *L. plantarum* 423 Fluc (Fig. 1b) and from approximately 1 × 10^7^ CFU to 2 × 10^9^ CFU for *E. mundtii* ST4SA Fluc (Fig. 1c).

No significant difference in growth was observed between wild-types (WT) *L. plantarum* 423 and *E. mundtii* ST4SA and recombinants *L. plantarum* 423 Fluc and *E. mundtii* ST4SA Fluc, respectively, after 9 h of growth (not shown). Bioluminescent light production and the presence of the luciferase expressing plasmids in recombinant *L. plantarum* 423 Fluc and *E. mundtii* ST4SA Fluc strains had no detectable effect on bacterial growth. The stability of the luciferase-expressing plasmids pNZPldhFfluc in *L. plantarum* 423 Fluc and pNZSTldhFfluc in *E. mundtii* ST4SA Fluc was tested in vitro by subculturing for up to 7 days with replica-plating on non-selective and selective media (Additional file 1: Figure S1a). The stability of the autonomous plasmids in *L. plantarum* 423 Fluc and *E. mundtii* ST4SA Fluc transformants was indicated by 100% plasmid retention and retained resistance to Cm following culturing for 7 days in the absence of the antibiotic. The bioluminescent signals of the recombinant strains were also imaged with the IVIS in parallel to replica plating (Additional file 1: Figure S1b and c).

### Colonization dynamics of bioluminescent *L. plantarum* 423 and *E. mundtii* ST4SA in the GIT of mice after a single dosage

**Fig. 2:**
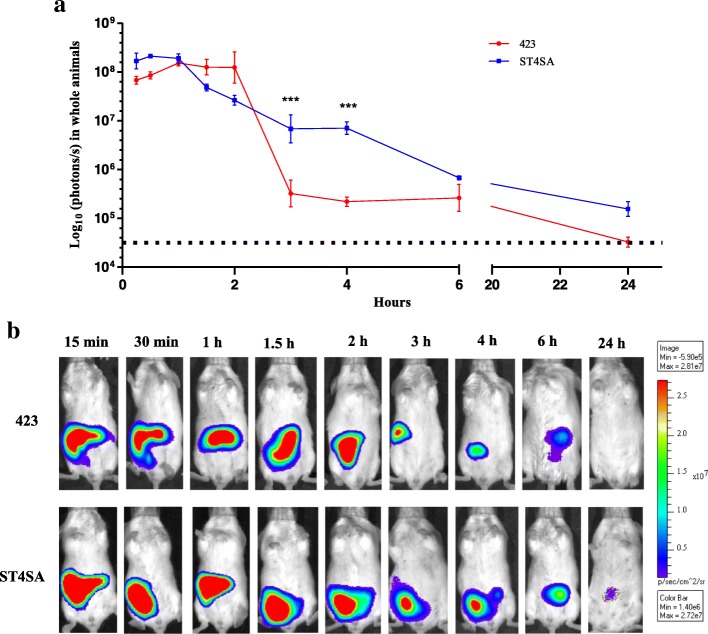
Monitoring of *L. plantarum* 423 and *E. mundtii* ST4SA colonization in the digestive tract of mice by bioluminescence imaging in whole animals after one oral administration. *Lactobacillus plantarum* 423 Fluc and *E. mundtii* ST4SA Fluc were fed intragastrically (4 × 10^9^ CFU) to two sets of three mice (*n* = 3, per strain). The bioluminescent signals in log_10_ photons/s measured from whole animals at different time-points over a 24 h period **(a)** are plotted, with standard deviations. Significant statistical differences between the bioluminescence signals of the two groups of mice are indicated with three asterisks (*P* < 0.001); Mann-Whitney nonparametric test). The background bioluminescence signal (approximately 4 × 10^4^ p/s) emitted is represented by a dashed line. **b** Visual images of bioluminescence emission in whole animals by mice fed once with *L. plantarum* 423 Fluc or *E. mundtii* ST4SA Fluc. In each case, one representative image of one mouse is shown. The intensity of the photon emission is represented as a pseudo-color image. One representative scale bar is shown (p/s)

To determine the spatial and temporal colonization of *L. plantarum* 423 Fluc and *E. mundtii* ST4SA Fluc after a single oral administration, groups of mice (*n* = 13, per strain) were monitored over a 24 h period by transcutaneous in vivo bioluminescence imaging (BLI) and ex vivo BLI of GITs and faeces. The viable bacteria numbers in the GIT and faecal samples were also recorded. Three anesthetised mice (*n* = 3, per strain) were imaged at 15 and 30 min and, 1, 1.5, 2, 3, 4, 6 and 24 h after the administration of *L. plantarum* 423 Fluc and *E. mundtii* ST4SA Fluc, respectively (Fig. 2). The same three mice were used throughout the 24 h trial period. At time zero (before administration) no bioluminescence was recorded (background signal corresponded to approximately 3 × 10^4^ p/s). A maximum bioluminescent signal of approximately 2 × 10^8^ p/s was detected for *L. plantarum* 423 Fluc and *E. mundtii* ST4SA Fluc at 1 h and 30 min, respectively. The bioluminescent signal of *L. plantarum* 423 Fluc remained at high levels until 2 h, but declined to lower levels 1 h later (mean value of approximately 9 × 10^6^ p/s). The bioluminescent signal of *L. plantarum* 423 Fluc remained at a plateau (mean value of approximately 4 × 10^5^ p/s) until 6 h. The bioluminescent signal of *E. mundtii* ST4SA Fluc steadily declined between 1 and 6 h and was significantly higher than that observed for *L. plantarum* 423 Fluc between 3 and 4 h. After 24 h, no bioluminescent signal could be detected for *L. plantarum* 423 Fluc (background level), whereas the signal of *E. mundtii* ST4SA Fluc declined to approximately 2 × 10^5^ p/s.

**Fig. 3:**
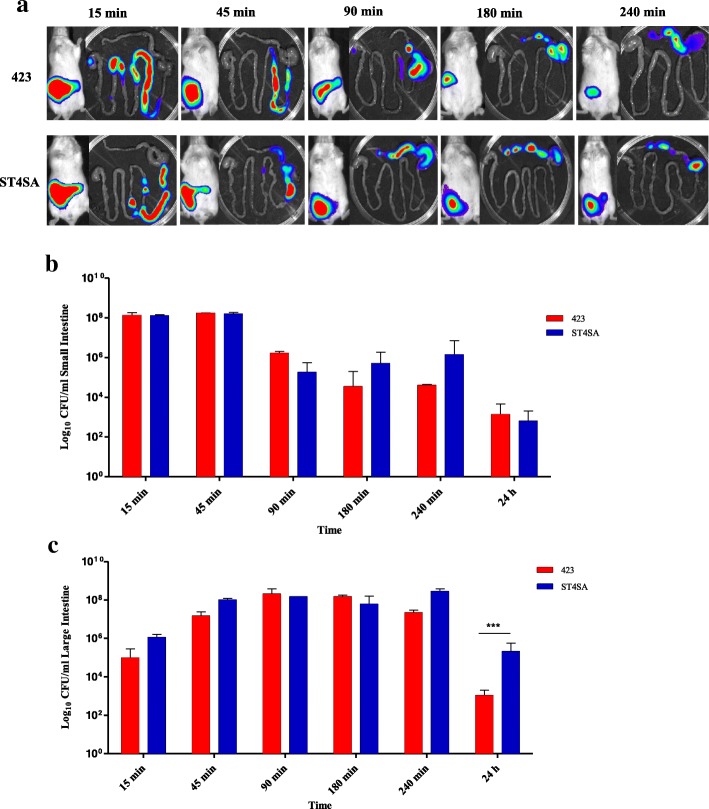
Transit of *L. plantarum* 423 and *E. mundtii* ST4SA through the digestive tract of mice after one oral administration. Groups of mice were gavaged once with 4 × 10^9^ CFU of *L. plantarum* 423 Fluc or *E. mundtii* ST4SA Fluc and the intestines resected at 15, 45, 90, 180, 240 min and 24 h. At each time point two mice (*n* = 12, per strain) were sacrificed, and **(a)** one representative image of one mouse and its GIT are shown. Persistence of viable *L. plantarum* 423 Fluc or *E. mundtii* ST4SA Fluc cells in **(b)** the small and **(c)** large intestinal tract of mice sacrificed at time points indicated in A. The limit of detection was approximately 4 × 10^4^ p/s. Significant differences between the two groups were assessed using the Mann-Whitney nonparametric test are indicated with asterisks (*P* < 0.001)

Next, the localization of bioluminescent *L. plantarum* 423 Fluc and *E. mundtii* ST4SA Fluc in the GITs of mice after oral administration was determined by ex vivo imaging and recording viable cell numbers in the small and large intestinal tracts (Fig. 3). Results showed that 15 min after administration of bacterial strains, both *L. plantarum* 423 Fluc and *E. mundtii* ST4SA Fluc survived passage through the stomach by the observation of high cell numbers and bioluminescent cells throughout the small intestine (Fig. 3a and b). After approximately 45 min, bioluminescent cells of *E. mundtii* ST4SA Fluc reached the cecum and colon. From 90 to 240 min after oral administration of bacteria to mice, the majority of bioluminescent *L. plantarum* 423 Fluc and *E. mundtii* ST4SA Fluc had travelled through the small intestine and were located exclusively in the cecum and colon. Some of the viable cells of both strains remained in the small intestine after 90 min, but emitted weak or no bioluminescence signals (Fig. 3b). No bioluminescence of either strain was detected in the large intestine 15 min (Fig. 3a) after intragastric administration, suggesting that the viable cells’ bioluminescence emission was below the detection limit of the IVIS through the intestinal tissue or the cells were metabolically inactive (Fig. 3c). After 24 h, a significantly higher number of viable *E. mundtii* ST4SA Fluc was detected in the cecum/colon compared to *L. plantarum* 423 Fluc.

**Fig. 4:**
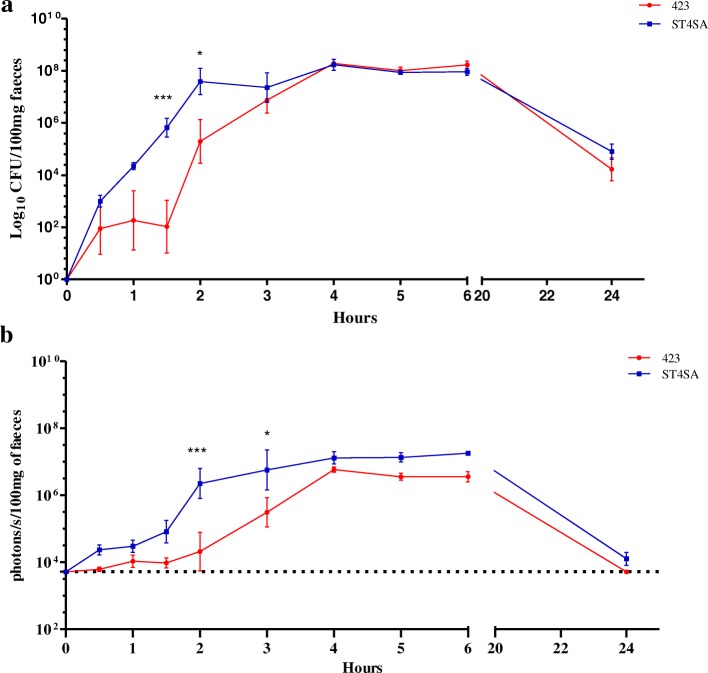
Presence of *L. plantarum* 423 and *E. mundtii* ST4SA in the faeces of mice after a single oral administration. Groups of three mice each (*n* = 3, per strain) were administered once with 4 × 10^9^ CFU *L. plantarum* 423 Fluc or *E. mundtii* ST4SA Fluc. At each time point, log_10_ averages of the **(a)** cell counts per 100 mg faeces and the corresponding **(b)** bioluminescence in log_10_ p/s per 100 mg faeces for each group of three mice are plotted with standard deviations. Significant differences between the two groups were assessed using the Mann-Whitney nonparametric test and are indicated with one (*P* < 0.05) or three (*P* < 0.001) asterisks. The background bioluminescence signal (approximately 5 × 10^3^ p/s) emitted is represented by a dashed line

Colonization of *L. plantarum* 423 Fluc and *E. mundtii* ST4SA Fluc in the GIT of mice was also determined by monitoring the number of viable bacterial cells in faeces at different time points after intragastric administration (Fig. 4a). The respective bioluminescent signals of *L. plantarum* 423 Fluc and *E. mundtii* ST4SA Fluc in faeces were also monitored with the IVIS **(**Fig. 4b). High cell numbers of both strains were excreted in the faeces and were proportionate to the respective bioluminescence signals emitted. The bacterial populations of both strains in faeces increased with time. Both strains reached a maximum number of approximately 2 × 10^8^ CFU/100 mg faeces after 4 h and remained at this level for the following 2 h. These peaks correlated excellently with maximum bioluminescent signals of approximately 4 × 10^6^ p/s/100 mg of faeces for *L. plantarum* 423 Fluc and 2 × 10^7^ p/s/100 mg of faeces for *E. mundtii* ST4SA Fluc, from 4 to 6 h. The maximum level of viable *E. mundtii* ST4SA Fluc cells shed in the faeces was significantly higher in the first 2 h, reaching approximately 8 × 10^7^ p/s/100 mg of faeces. Bioluminescence signals emitted by *E. mundtii* ST4SA Fluc cells in the faeces were higher compared to those of *L. plantarum* 423 Fluc throughout the 24 h study period. After 24 h, the number of *L. plantarum* 423 Fluc declined to approximately 4 × 10^4^ CFU/100 mg faeces with no bioluminescent signal (background), while *E. mundtii* ST4SA Fluc cells declined to approximately 1 × 10^5^ CFU/100 mg faeces with a weak bioluminescent signal of 1.5 × 10^4^ p/s/100 mg of faeces.

### Persistence of bioluminescent *L. plantarum* 423 and *E. mundtii* ST4SA in the GIT of mice after five oral dosages

**Fig. 5:**
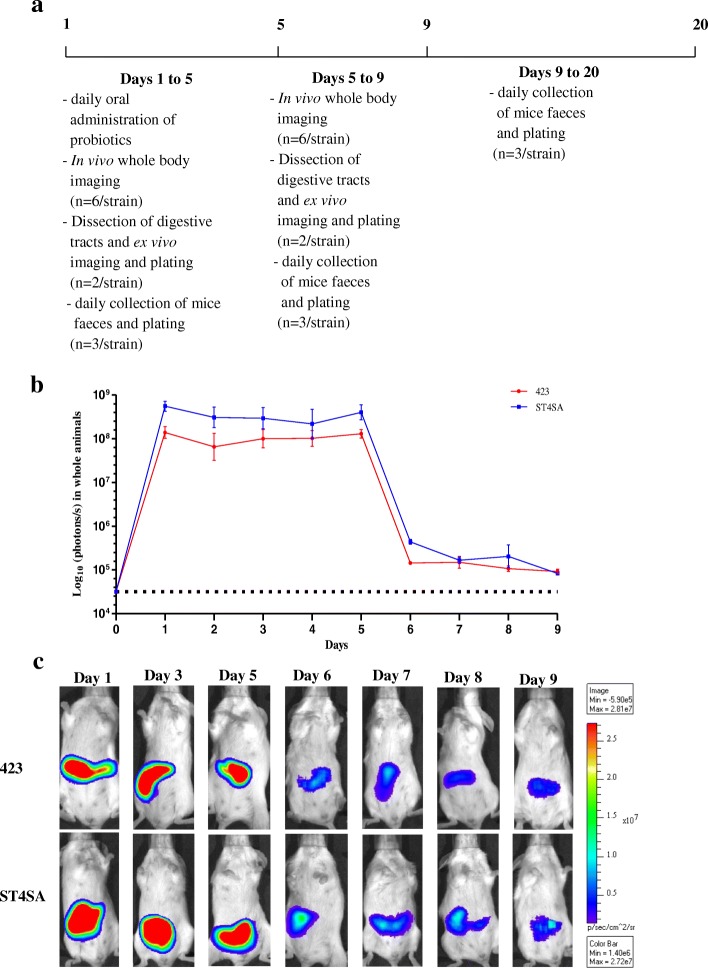
Monitoring of colonization and persistence of *L. plantarum* 423 and *E. mundtii* ST4SA in the GIT of mice by bioluminescence imaging in whole animals after five daily administrations. **a** The experimental design. *Lactobacillus plantarum* 423 Fluc and *E. mundtii* ST4SA Fluc were fed once daily by intragastric gavage (4 × 10^9^ CFU) to two groups of mice (*n* = 22, per strain) for five consecutive days (days 1 to 5). The bioluminescent signals in log_10_ photons/s measured in whole animals from day 1 to 9 for each set of three mice **(b)** are plotted, with standard deviations. No statistical differences between the bioluminescence signals of the two groups of mice were observed. The background bioluminescence signal (approximately 4 × 10^4^ p/s) emitted is represented by a dashed line. **c** Visual images of bioluminescence emission in whole animals by mice fed once daily for five consecutive days with *L. plantarum* 423 Fluc or *E. mundtii* ST4SA Fluc. One representative image of one mouse is shown. The intensity of the photon emission is represented as a pseudo-color image. One representative scale bar is shown (p/s)

In vivo bioluminescent signals were measured everyday (1 h after administration of bacteria to mice on days 1 to 5) for nine days after two groups of mice (*n* = 22, per strain) each received daily doses of *L. plantarum* 423 Fluc and *E. mundtii* ST4SA Fluc for five consecutive days (Fig. 5). The experimental design is described in Fig. 5a. High intensity bioluminescence was emitted by mice in both groups from days 1 to 5. Highest bioluminescent signals were recorded for *E. mundtii* ST4SA Fluc (mean = 4.27 × 10^8^ p/s) compared to *L. plantarum* 423 Fluc (mean = 7.78 × 10^7^ p/s) during the first 5 days. After 6 days the signal for *L. plantarum* 423 Fluc rapidly declined to approximately 1 × 10^5^ p/s, while the signal for *E. mundtii* ST4SA Fluc declined to approximately 5 × 10^5^ p/s. From day 6, the *L. plantarum* 423 Fluc bioluminescent signal remained at a plateau until day 9 (approximately 1 × 10^5^ p/s). The *E. mundtii* ST4SA Fluc bioluminescent signal declined to approximately 2 × 10^5^ p/s on day 7 and to approximately 8 × 10^4^ p/s after 9 days.

**Fig. 6:**
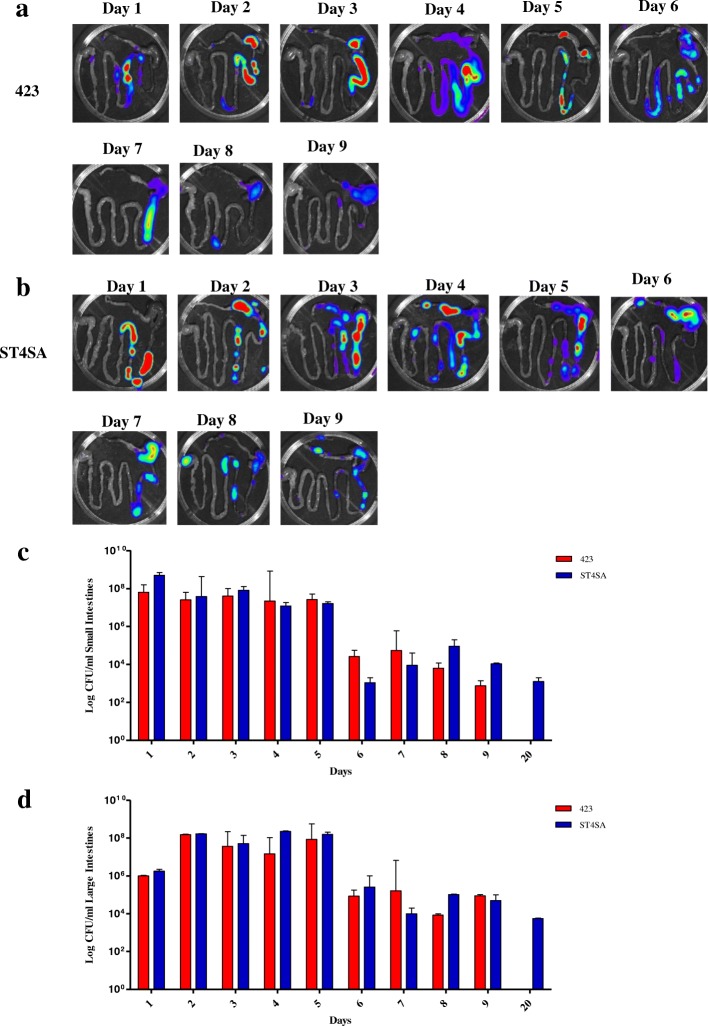
Comparison of colonization abilities of *L. plantarum* 423 and *E. mundtii* ST4SA in the GIT of mice after five daily oral administrations. *Lactobacillus plantarum* 423 Fluc and *E. mundtii* ST4SA Fluc were fed once daily by oral gavage (4 × 10^9^ CFU) to two groups of mice (*n* = 18, per strain) for five consecutive days (days 1 to 5). Four mice (two per group) were sacrificed from day 1 to 9, and a representative image of the GIT of one mouse is shown (days 1 to 9) in mice fed with **(a)** *L. plantarum* 423 Fluc or **(b)** *E. mundtii* ST4SA Fluc. Persistence of viable *L. plantarum* 423 Fluc or *E. mundtii* ST4SA Fluc cells in **(c)** the small and **(d)** large intestinal tract of mice sacrificed at time points indicated in A and B and day 20. The limit of detection was approximately 4 × 10^4^ p/s

The intestines of mice were imaged ex vivo to determine which sections of the GIT the bacterial strains colonized after 5 oral administrations (Fig. 6). On day 1, both strains were detected throughout the small intestine 30 min after oral administration of bacteria. From day 2 to day 5, both strains were detected in the jejunum and ileum sections of the small intestine (jejunum and ileum) and in both the cecum and colon sections of the large intestinal tract. At days 6 and 7 (1 and 2 days after last bacterial dose), *L. plantarum* 423 Fluc colonized the small intestine and cecum readily, but the bioluminescent signal detected from the small intestine progressively declined and by day 9 a very weak or no bioluminescent signal remained (Fig. 6a). On day 9, *L. plantarum* 423 Fluc was predominantly localized in the cecum and colon. From day 6 to day 9, *E. mundtii* ST4SA Fluc colonized the upper section of the small intestine and the cecum/colon most prominently (Fig. 6b).

Viable counts of the small and large intestine taken on days 1 to 5 revealed high numbers (approximately 10^8^ CFU) of both *L. plantarum* 423 Fluc and *E. mundtii* ST4SA Fluc (Fig. 6c and d). At day 7 (2 days after last bacterial dose), the colonization of the large intestine (mean value of 3 × 10^5^ CFU) by *L. plantarum* 423 Fluc was 10-fold superior to that of the small intestine (mean value of 3 × 10^6^ CFU). Two days later, the difference between the amount of viable *L. plantarum* 423 Fluc cells in the small (mean value of 9 × 10^2^ CFU) and large intestine (mean value of 8 × 10^4^ CFU) increased to 100-fold. In the case of *E. mundtii* ST4SA Fluc, the number of viable cells in the small intestine increased from approximately 1 × 10^3^ CFU on day 6 to 2 × 10^4^ CFU on day 9. On day 9, mice administered with *L. plantarum* 423 Fluc harboured approximately 9 × 10^4^ CFU in the cecum/colon, while mice administered *E. mundtii* ST4SA Fluc harboured approximately 7 × 10^4^ CFU in the cecum/colon. After 20 days, no *L. plantarum* 423 Fluc were detected in the intestines of mice. However, the number of *E. mundtii* ST4SA Fluc cells in the small intestine was still approximately 1.4 × 10^3^ CFU and 5 × 10^3^ CFU in the large intestine.

### Persistence of *L. plantarum* 423 and *E. mundtii* ST4SA in faeces after five oral dosages

**Fig. 7:**
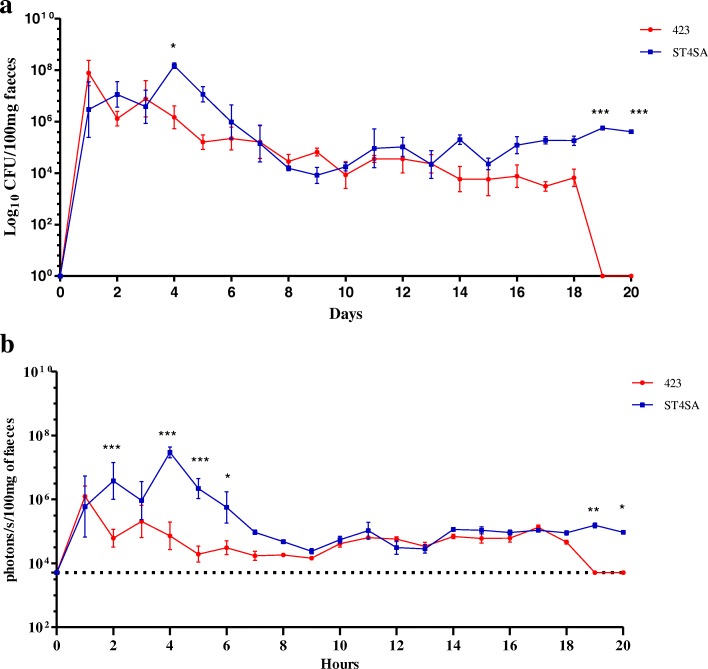
Persistence of *L. plantarum* 423 and *E. mundtii* ST4SA in mouse faeces after five daily oral administrations. Groups of three mice (n = 3, per strain) each were administered once daily (days 1 to 5) with 4 × 10^9^ CFU *L. plantarum* 423 Fluc or *E. mundtii* ST4SA Fluc for five consecutive days. Mouse faeces were collected daily from days 1 to 20. At each time point, log_10_ averages of the **a** cell counts per 100 mg faeces and the corresponding **b** bioluminescence in log_10_ p/s per 100 mg faeces for each group of three mice are plotted with standard deviations. Significant differences between the two groups were assessed using the Mann-Whitney nonparametric test and are indicated with one (*P* < 0.05), two (*P* < 0.01) or three (*P* < 0.001) asterisks. The background bioluminescence signal (approximately 5 × 10^3^ p/s) emitted is represented by a dashed line

The persistence of bioluminescent *L. plantarum* 423 Fluc and *E. mundtii* ST4SA Fluc in faeces and their respective bioluminescent signals were monitored every day for 20 consecutive days (Fig. 7a and b). *Enterococcus mundtii* ST4SA Fluc persisted in the faeces throughout the trial, with significantly higher cell numbers recorded at days 4, 19 and 20 compared to *L. plantarum* 423 Fluc. The maximum number of viable *E. mundtii* ST4SA Fluc cells in faeces was excreted at day 4 (approximately 2 × 10^8^ CFU/100 mg faeces). From day 4 to 9 the level of *E. mundtii* ST4SA Fluc cells in faeces declined to approximately 1 × 10^4^ CFU/100 mg faeces. After day 9 there is a slight increase in the number of *E. mundtii* ST4SA Fluc cells in faeces until day 11 after which levels of approximately 10^4^ to 10^5^ CFU/100 mg faeces were maintained from days 12 to 20. *Lactobacillus plantarum* 423 Fluc, on the other hand, reached a maximum level of approximately 2 × 10^8^ CFU/100 mg faeces at day 1 and then steadily declined until day 19 (mean value of 1 × 10^4^ CFU/100 mg faeces). *Lactobacillus plantarum* 423 Fluc persisted for only 13 days in faeces after the last day of intragastric administration of bacteria (day 5).

The bioluminescent signal of *E. mundtii* ST4SA Fluc cells in faeces was detected throughout the trial period, while the *L. plantarum* 423 Fluc bioluminescent signal declined to the background level at day 19. The peaks of the amount of viable cells per 100 mg faeces of both strains correlated perfectly with the amount of bioluminescent signals emitted at different time points. The bioluminescent signal of *L. plantarum* 423 Fluc was detected at lower levels between days 2 and 6, but displayed levels similar to those observed for *E. mundtii* ST4SA Fluc from day 7 until 18. The bioluminescent system allowed the detection of both *L. plantarum* 423 Fluc and *E. mundtii* ST4SA Fluc viable cells in faeces as low as 10^4^ CFU/100 mg faeces.

## Discussion

A major advantage of using the BLI technique compared to conventional approaches is that it allows for drastic reductions in the number of animals to be sacrificed to establish the precise location of bacteria in mouse or rat models [20]. Moreover, more information is gathered over a shorter period per experiment compared to traditional pre-clinical animal trials [21]. Mouse models provide a complex whole body system for the non-invasive real-time monitoring of bioluminescent probiotic LAB through the GIT of the mammalian host [43]. The murine model is the predominant choice for the in vivo evaluation of probiotic properties and has been used to study the persistence and localization of potential probiotic LAB in several studies [21, 24, 27, 33, 43]. In the current study, an IVIS system and the BLI technique were used to study the colonization dynamics of the probiotic LAB strains *L. plantarum* 423 and *E. mundtii* ST4SA in the GIT of mice.

The stable expression of reporter genes during in vivo imaging is an absolute prerequisite for the detection of luminescent light through animal tissues. Stable expression of reporter genes depends on the nature of the expression vector used, including the promoters used to drive expression and plasmid copy numbers. With this in mind, expression of the firefly luciferase gene from *P. pyralis* (*ffluc*) was optimized for use in *L. plantarum* 423 and *E. mundtii* ST4SA. Results in this study demonstrate that highest bioluminescence signals were achieved using the pNZ8048 high-copy number plasmid [44] for luciferase gene expression (Fig. 1). Attempts to use a low-copy number plasmid and chromosomally integrated *ffluc* genes in *L. plantarum* 423 and *E. mundtii* ST4SA resulted only in the emission of weak and inconsistent bioluminescent signals (not shown). The former results are most likely linked to the presence of multiple copies of the pNZ8048 plasmid that in turn leads to higher expression levels of the *ffluc* gene within the respective LAB hosts. The expression of reporter genes in bacteria using plasmids is widely used. Bacterial plasmids play an important role in the ability of bacteria to adapt in diverse environments [45]. However, when introducing a new recombinant plasmid harbouring a reporter gene, it is critical to evaluate its stability and persistence within the bacterial host with and without antibiotic pressure. This study demonstrates that in vitro *ffluc* expression plasmids were remarkably stable in *L. plantarum* 423 Fluc and *E. mundtii* ST4SA Fluc and that bioluminescence did not affect the growth of the respective host strains compared to the WT derivatives (see Additional file 1). We have previously demonstrated plasmid stability in *E. mundtii* ST4SA [24]. In terms of promoter selection, the lactate dehydrogenase gene (*ldh*) promoters of *L. plantarum* 423 and *E. mundtii* ST4SA, respectively, drove highest *ffluc* gene expression. The *L. plantarum ldh* gene promoter has been used for the constitutive expression of genes in LAB in several studies [24, 33, 46, 47]. These results demonstrated that both *L. plantarum* 423 Fluc and *E. mundtii* ST4SA Fluc could produce bioluminescence with no loss of the firefly luciferase-expressing plasmids.

The current study was designed to study the colonization (spatial and temporal) dynamics of *L. plantarum* 423 and *E. mundtii* ST4SA orally inoculated once or for five consecutive days over a period of 24 h and 20 days, respectively. Strong bioluminescence signals emitted by *L. plantarum* 423 Fluc and *E. mundtii* ST4SA Fluc could be detected as soon as 15 min after oral administration with a single bacterial dosage of the respective strains (Fig. 2). The bioluminescence signals of both strains as recorded with whole body imaging showed an overall decline after a single oral administration to mice. This is an indication that the probiotic strains only transiently colonized the GIT of mice after a single oral administration. In a previous report, using the *mCherry* fluorescence gene and a conventional culture-based method, we showed similar transit dynamics of viable *L. plantarum* 423 and *E. mundtii* ST4SA after a single dose of each bacterial strain [24]. However, we could not obtain fluorescence signals from in vivo whole body imaging for real-time analysis and could only locate the bacterial strains in the GIT after killing the animals. Interestingly, in the current study bioluminescence signals were detected up to 6 h in whole animals fed with either *L. plantarum* 423 or *E. mundtii* ST4SA and up to 24 h in mice fed with *E. mundtii* ST4SA. Eom et al. [27] could not detect a bioluminescence signal after 3 h from mice administered bioluminescent *L. casei* CJNU 0588. Based on whole body bioluminescence imaging in real-time, these results clearly reflect the superior active replication and colonization abilities of *L. plantarum* 423 and *E. mundtii* ST4SA in the mouse GIT compared to *L. casei* CJNU 0588.

The bacterial transit time in the GIT for viable *L. plantarum* 423 and *E. mundtii* ST4SA varied. *Lactobacillus plantarum* 423 showed a slower transit and was detected in the lower part of the jejunum and in the ileum 45 min post gavage, while *E. mundtii* ST4SA was detected in the ileum, cecum and colon (Fig. 3a). Similarly, Karimi et al. [48] demonstrated that the gastrointestinal transit time differs between strains of *L. reuteri*, using fluorescence and bioluminescence imaging techniques. However, the authors did not selectively monitor the bacterial cell numbers of the LAB strains present in gastrointestinal tissue or stool samples. Although it was shown in the current study that there is excellent correlation between bioluminescence and bacterial load, it is important to verify that a certain level of bioluminescence correlates to a certain number of cells. Enumeration of viable counts in dissected intestines revealed that both bioluminescent LAB strains were located predominantly in the large intestine (cecum/colon) 3 h post gavage (Fig. 3b). No bioluminescent cells of *L. plantarum* 423 or *E. mundtii* ST4SA were detected in the stomach or duodenum compartments 45 min after intragastric administration of the respective bacteria (Fig.3a). These results are indicative of the harsh conditions the LAB strains are exposed to in those compartments of the GIT and may be the reason for the rapid transit in those sections of the gut. The CFU counts revealed the presence of viable cells of *L. plantarum* 423 and *E. mundtii* ST4SA in the small intestine at 4 and 24 h post gavage, but no bioluminescent signals could be detected. This might be caused by reduced cell activity or the inhibition of protein synthesis during passage in the stomach and duodenum compartments of the gut, which leads to inefficient bioluminescence emission. Bioluminescent light can only be produced in metabolically active cells [20]. Both strains could be detected in the cecum and colon after 4 h, indicating the metabolically active state of the bacteria in the large intestine (Fig. 3a).

After 24 h, no bioluminescent signal could be detected from mice administered with *L. plantarum* 423, while a detectable bioluminescent signal was still observed from mice fed with *E. mundtii* ST4SA (Fig. 2). This indicates that *L. plantarum* 423 was eliminated from the GIT of mice more rapidly than *E. mundtii* ST4SA, and is associated with a significantly higher amount of viable *E. mundtii* ST4SA in the large intestine compared to *L. plantarum* 423 (Fig. 3c). These findings are in agreement with previous studies [33, 48] reporting on the gastrointestinal transit of *Lactobacillus reuteri, L. plantarum* and *Lactococcus lactis.* The authors showed that the intestinal transit times differed between two strains of *L. reuteri,* and that a *Lc. lactis* strain had shorter survival times in the GIT compared to a *L. plantarum* strain. *Lactobacillus plantarum* 423 and *E. mundtii* ST4SA were excreted in high numbers in the faeces of mice over the 24 h period, but it is clear that a small amount of each LAB strain persisted in the GIT after a single oral dose as demonstrated with intestinal tissue CFU counts (Figs. 3 and 4). Bioluminescence signals from bacteria in faecal material accurately reflected CFU data and this serves as an indication that the plasmids are retained in vivo to a large extent.

To study more thoroughly the persistence of the bioluminescent strains in the GIT, mice were administered orally with either *L. plantarum* 423 or *E. mundtii* ST4SA for five consecutive days. Our results demonstrate that *L. plantarum* 423 and *E. mundtii* ST4SA had similar GIT transit dynamics during the first 5 days of administration of the respective strains to mice, despite the detection of higher bioluminescent signals for *E. mundtii* ST4SA compared to *L. plantarum* 423 (Fig. 5). At day 6 (one day after last bacterial dosage), the in vivo bioluminescent signals of both strains declined to low levels but were maintained at similar levels for the next 3 days. This suggests that while most of the administered bacteria transited the GIT of mice, small populations of both strains persisted until day 9 (4 days after last bacterial dosage). Overall, the gastrointestinal persistence of *L. plantarum* 423 and *E. mundtii* ST4SA compared well to several other commercial probiotic strains, including *Lactobacillus rhamnosus* GG, *Bifidobacterium lactis* LAFTI B94, *L. plantarum* 299v and *Lactobacillus gasseri* SBT2055 [49–52]. These observations were confirmed by ex vivo imaging of dissected intestines (Fig. 6a and b). At day 9, bioluminescent cells of both strains were predominantly localized in the cecum and colon. It has been suggested that the murine cecum may be the site where microorganisms adapt to the gastrointestinal environment and where the activation of genes required for colonization of the colon occur [29]. Interestingly, the cecum and colon have also been shown to be the major sites of colonization of several enteric pathogens including *E. coli* O157:H7, *Citrobacter rodentium*, *Yersinia enterocolitica*, *L. monocytogenes* and *S. typhimurium* [42, 53–56]. Both *L. plantarum* 423 and *E. mundtii* ST4SA have been demonstrated to exclude pathogens such as *L. monocytogenes*, *S. typhimurium, C. sporogenes* and *E. faecalis* in in vitro or in vivo competitive exclusion experiments [40–42]. The presence of persistent populations of *L. plantarum* 423 and *E. mundtii* ST4SA in the murine cecum/colon as demonstrated in this study suggest that their presence may have prevented the pathogenic bacteria from becoming established. It is also interesting to note that the population of *E. mundtii* ST4SA in the small intestine increased until day 9 compared to that of *L. plantarum* 423 (Fig. 6c). This could explain why mice pre-colonized with *E. mundtii* ST4SA showed a more rapid decline in *L. monocytogenes* EGDe cell numbers compared *L. plantarum* 423 in a competitive exclusion experiment [42]. *Enterococcus mundtii* ST4SA cells were able to persist in the faeces of mice throughout the trial period (Fig. 7). In contrast, *L. plantarum* 423 could not be detected in faeces after 13 days after the last oral administration to mice and in lower numbers compared to *E. mundtii* ST4SA. Based on bioluminescence, the amount of bacteria administered to mice per day (4 × 10^9^ CFU) and the amount of *E. mundtii* ST4SA cells shed in the faeces per day, there is a clear indication that *E. mundtii* ST4SA persists better than *L. plantarum* 423 in the murine GIT of mice. Since the same dosage and administration methods were used for both strains, the difference in the intestinal persistence between the two probiotic strains might be due to differences in the physiological and genotypic properties of the strains.

## Conclusions

The construction and optimization of LAB reporter strains is an important step towards a better understanding of the route and destination of orally administered probiotics in the GIT, and the interactions between probiotics and the host. This study demonstrates the application of the firefly luciferase system to compare the colonization dynamics of *L. plantarum* 423 and *E. mundtii* ST4SA in mice. The in vivo BLI system revealed the precise location of the bacterial strains within the murine GIT after single or multiple doses. Both strains prominently colonized the cecum and colon. *Enterococcus mundtii* ST4SA persisted in the GIT and faeces of mice throughout the trial period and also actively colonized the small intestine. This is the first report of bioluminescence in vivo imaging of *E. mundtii* ST4SA in a mouse model. The bioluminescence system developed here has the potential to allow the study of in vivo colonization dynamics of other important probiotic LAB species.

## Methods

### Bacterial strains, plasmid construction and culture conditions

*Escherichia coli* MC1061 (Mobitech) was used as a cloning host for construction of pNZ8048-derived bioluminescence expression vectors and was cultured aerobically at 37 °C in Luria-Bertani (LB) broth, or brain heart infusion (BHI) and streaked onto the same media, supplemented with 1.5% (*w*/*v*) agar (all from Biolab Diagnostics, Midrand, South Africa). *Lactobacillus plantarum* 423 and *E. mundtii* ST4SA were grown without shaking at 30 °C in MRS broth and streaked onto MRS agar (both from Biolab Diagnostics). Where appropriate, Cm was added at 10 μg/ml to growth media of *E. coli* MC1061 and *L. plantarum* 423 and 5 μg/ml to media of *E. mundtii* ST4SA.

*Lactobacillus plantarum* 423 and *E. mundtii* ST4SA were labelled by transformation with plasmids encoding the red-shifted thermostable firefly luciferase gene from *P. pyralis* (*ffluc*) [57]. The bioluminescence expression vectors are based on the pNZ8048 *Lc. lactis* NICE system high copy number plasmid (Mobitech, Goettingen, Germany). The vector contains the *cat* gene for chloramphenicol (Cm) resistance, the *nisA* gene promoter region (P*nisA*), a multiple cloning site (MCS), replication genes (*repC* and *repA*) for replication in LAB/*E. coli* and the termination (T) sequence of the *Lc. lactis pepN* gene [44]. Primers used for PCR amplification are listed in Additional file 2: Table S1 and were from Inqaba Biotechnical Industries (Pretoria, South Africa). DNA restriction enzymes and PCR polymerase were from New England Biolabs (NEB, Ipswich, MA, USA). The construction of pNZPldhFfluc and pNZSTldhFfluc luciferase expression vectors is shown in Additional file 3: Figure S2. Plasmid pNZPldhFfluc carried the *ffluc* gene under control of the strong constitutive *L. plantarum* 423 lactate dehydrogenase gene promoter (P*ldh*). In the pNZSTldhFfluc construct, the *ffluc* gene was cloned under control of the strong constitutive *E. mundtii* ST4SA lactate dehydrogenase gene promoter (ST*ldh*). The P*ldh* and ST*ldh* promoters were amplified from *L. plantarum* 423 and *E. mundtii* ST4SA genomic DNA, using primer pairs Pldh1/Pldh2 and ldhS1/ldhS2, respectively. The *ffluc* bioluminescence gene was amplified from plasmid pMV306G13 + FflucRT using primers FlucFor and FlucRev. Briefly, the P*ldh* (520 bp), ST*ldh* (166 bp) and *ffluc* (1.6 kb) PCR fragments were cloned into pNZ8048 after digestion of P*ldh* and ST*ldh* with *Bgl*II/*Nco*I, digestion of *ffluc* with *Nco*I/*Xba*I and digestion of pNZ8048 with *Bgl*II/*Xba*I (resulting in the removal of the PnisA promoter), yielding plasmids pNZPldhFfluc and pNZSTldhFfluc, respectively.

The two bioluminescence expression vectors were introduced into *L. plantarum* 423 and *E. mundtii* ST4SA by electro-transformation as described by Van Zyl et al. [24] and were named *L. plantarum* 423 Fluc and *E. mundtii* ST4SA Fluc. *Lactobacillus plantarum* 423 and *E. mundtii* ST4SA containing the empty pNZ8048 vector were used as controls and were labelled *L. plantarum* 423 (pNZ8048) and *E. mundtii* ST4SA (pNZ8048). Plasmid stability in *L. plantarum* 423 Fluc and *E. mundtii* ST4SA Fluc, and growth comparison between WT and recombinant strains was tested by standard methodology as described previously [24].

### Correlation between in vitro bioluminescence measurements and viable cell numbers

*Lactobacillus plantarum* 423 Fluc and *E. mundtii* ST4SA Fluc were grown for 12 h at 30 °C in MRS broth, supplemented with Cm as mentioned elsewhere. From these cultures, 1 ml was inoculated into freshly prepared MRS broth and incubated at 30 °C to an optical density (OD_550nm_) of 2.5 (for *L. plantarum* 423) and 2.3 (for *E. mundtii* ST4SA). Viable cell numbers were determined by plating onto MRS agar containing Cm. The bacterial suspensions were harvested (3 min at 8000 x g), washed twice with sterile PBS, resuspended in gavage buffer (0.2 M NaHCO_3_ with 1%, *w*/*v*, glucose, pH 8.0) and serially diluted to 1/128 in the same buffer. Two-hundred microliters of each dilution was added in triplicate to black 96-well microtitre plates and bioluminescence measured after the addition of 5 μl of D-Luciferin potassium salt (Anatech Instruments, Bellville, South Africa) at 470 μM. Bioluminescent readings were recorded using the IVIS and the photons emitted from regions of interest (ROI) calculated using the Living Image® software, version 3.0 (Caliper Life Sciences). The ROI of each well were manually selected. Exposure times ranged from 30 s to 2 min, depending on the intensity of the signal. Bacterial cell numbers were plotted against bioluminescence emitted, recorded as p/s. Non-bioluminescent *L. plantarum* 423 (pNZ8048) and *E. mundtii* ST4SA (pNZ8048) were used to set the background bioluminescence. A modified version of the method by Rhee et al. [58], was used for the in vitro plasmid stability experiment.

### Animals used

Ethical approval for in vivo experiments was granted by the Ethics Committee of Stellenbosch University (reference number SU-ACU-2017-0206-454). Eight-week-old female BALB/c mice were used in all experiments and were obtained from South African Vaccine Producers (Pty.) Ltd. (Sandringham, Pretoria, South Africa). Animals were housed in separate cages under controlled environmental conditions (12 h dark/light cycles, 20–22 °C). Water and a standard rodent feed was provided ad libitum and changed daily. Animal procedures were performed according to the Stellenbosch University ethical guidelines.

### Preparation of bacterial strains and dosing of mice

*Lactobacillus plantarum* 423 Fluc and *E. mundtii* ST4SA Fluc were grown at 30 °C for 12 h, whereafter 1 ml of each culture was inoculated into freshly prepared 10 ml MRS broth. *Lactobacillus plantarum* 423 Fluc was grown to an OD_550_ of 2.5 and *E. mundtii* ST4SA Fluc to an OD_550_ of 2.3. The cells were harvested (3 min at 8000 x g), washed twice with sterile PBS and resuspended in gavage buffer at a final concentration of 4 × 10^9^ CFU. Mice in each group were then gavaged with 200 μl (4 × 10^9^ CFU) of each strain.

### In vivo gastrointestinal persistence of LAB in the murine model

Groups of mice each received a daily dose of 200 μl (4 × 10^9^ CFU) of live *L. plantarum* 423 Fluc or *E. mundtii* ST4SA Fluc for one (*n* = 13, per strain) or five (*n* = 22, per strain) consecutive days by intragastric gavage. Control mice (*n* = 4, per strain) received 200 μl (4 × 10^9^ CFU) of non-bioluminescent *L. plantarum* 423 (pNZ8048) or *E. mundtii* ST4SA (pNZ8048) in all experiments. Faeces (100 mg) were collected at different time points and vortexed in 1 ml sterile PBS for 5 min, followed by serial dilution in sterile PBS, and plating onto MRS agar supplemented with Cm and incubated, as described elsewhere. Viable cell numbers were expressed as CFU per 100 mg faeces. Two mice per strain were sacrificed by cervical dislocation at predetermined time points, the intestines surgically removed and immediately separated in a sterile Petri dish. The lumen of all intestinal sections was injected with air using a 27-gauge needle and syringe, as described by Rhee et al. [58], and bioluminescence recorded using the IVIS. The complete duodenum, jejunum, ileum and large intestine (cecum plus colon) were homogenized, separately, in 3 ml sterile PBS, serially diluted and plated (in duplicate) onto MRS agar supplemented with Cm as mentioned elsewhere. The plates were incubated and cell numbers determined as described elsewhere.

### In vivo bioluminescence measurements

In vivo BLI was recorded using the IVIS, equipped with a cooled-charged-device camera mounted on a light-tight specimen chamber (dark box) and a Windows computer system. Mice were gavaged with 200 μl of a D-Luciferin potassium salt suspension (30 mg/ml) 30 min before gavage with *L. plantarum* 423 Fluc and *E. mundtii* ST4SA Fluc. Mice were anesthetized with 2% (vol/vol) isoflurane in an oxygen-rich induction chamber before administering the D-Luciferin and bacteria. Mice were kept subdued during bioluminescent readings with a mixture of isoflurane (1.5%, vol/vol) and oxygen. Mice in ventral position were imaged for quantification of bioluminescent photon emission with exposure times ranging from 1 to 5 min, depending on the signal intensity. Pseudo-color images superimposed over grayscale reference images representing light intensity (red, most intense and purple being the least intense) were generated using the Living Image® software program. ROIs were manually selected and bioluminescence expressed as photons emitted per second.

## Statistical analysis

All data were analysed using GraphPad Prism (version 6.05) and statistical differences between groups were determined using the Mann-Whitney nonparametric test. Statistical differences are shown for each data set. Error was calculated as standard error of mean (SEM). The number of animals required for each experiment was calculated (power analysis) using the resource equation for the sample size.

## Additional files

Additional file 1: **Figure S1.** In vitro stability of bioluminescence. (a) Stability of plasmid pNZPldhFfluc in *L. plantarum* 423 Fluc and plasmid pNZSTldhFfluc in *E. mundtii* ST4SA Fluc after subculturing for 7 days with replica plating on non-selective (antibiotic-free) and selective (Cm) media. The percentages of Cm-resistant colonies of three independent cultures of each respective strain are shown. Bioluminescent colonies of (b) *L. plantarum* 423 Fluc and (c) *E. mundtii* ST4SA Fluc after 7 days of subculture in antibiotic-free MRS media. (PDF 515 kb)

Additional file 2: **Table S1.** Primers used in this study. (PDF 497 kb)

Additional file 3: **Figure S2.** Schematic representing the construction of the pNZPldhFfluc and pNZSTldhFfluc luciferase expression plasmids. Relevant features are indicated, including restriction sites and PCR primers used for cloning; the *E. coli*/LAB *repA* and *repC* replication genes; the chloramphenicol acetyltransferase (*cat*) gene conferring resistance to chloramphenicol; the P*ldh* promoter from the *L. plantarum* 423 lactate dehydrogenase gene and the ST*ldh* promoter from the *E. mundtii* ST4SA lactate dehydrogenase gene. (PDF 507 kb)

## Abbreviations

BLI: Bioluminescence imaging
CBluc: Click beetle luciferase
CFU: Colony forming units
Cm: Chloramphenicol
DNA: Deoxyribonucleic acid
Ffluc: Firefly luciferase
GIT: Gastrointestinal tract
IVIS: In vivo imaging system
LAB: Lactic acid bacteria
LB: Luria Bertani
MCS: Multiple cloning site
OD: Optical density
p/s: Photons per second
PBS: Phosphate buffered saline
P*ldh*: Lactate dehydrogenase gene promoter of *Lactobacillus plantarum* 423
r*epA*: Replication gene A
*repC*: Replication gene C
ROI: Region of interest
ST*ldh*: Lactate dehydrogenase gene promoter of *Enterococcus mundtii* ST4SA
T: Termination sequences

## References

[1] Jens W (2008). Ecological role of lactobacilli in the gastrointestinal tract: implications for fundamental and biomedical research. Appl Environ Microbiol..

[2] de Vos WM (2011). Systems solutions by lactic acid bacteria: from paradigms to practice. Microb. Cell Fact..

[3] Marco ML, Pavan S, Kleerebezem M (2006). Towards understanding molecular modes of probiotic action. Curr Opin Biotechnol..

[4] Reid G (2005). The importance of guidelines in the development and application of probiotics. Curr Pharm Des..

[5] Sanz Y, Nadal I, Sánchez E (2007). Probiotics as drugs against human gastrointestinal infections. Recent Pat Antiinfect Drug Discov..

[6] Chmielewska A, Szajewska H (2010). Systematic review of randomised controlled trials: probiotics for functional constipation. World J Gastroenterol..

[7] Narayan SS, Jalgaonkar S, Shahani S, Kulkarni VN (2010). Probiotics: current trends in the treatment of diarrhoea. Hong Kong Med J..

[8] Tsai YT, Cheng PV, Pan TM (2012). The immunomodulatory effects of lactic acid bacteria for improving immune functions and benefits. Appl Microbiol Biotechnol..

[9] De ADM LB, Matar C, Perdigón G (2007). The application of probiotics in cancer. Brit J Nutr..

[10] Riaz Rajoka MS, Shi J, Zhu J, Shao D, Huang Q, Yang H (2017). Capacity of lactic acid bacteria in immunity enhancement and cancer prevention. Appl Microbiol Biotechnol..

[11] LeBlanc JG, Milani C, de Giori GS, Sesma F, van Sinderen D, Ventura M (2013). Bacteria as vitamin supplier to their host: a gut microbiota perspective. Curr Opin Biotechnol..

[12] Bezkorovainy A (2001). Probiotics: determinants of survival and growth in the gut. Am J Clin Nutr..

[13] Douillard FP, de Vos WM (2014). Functional genomics of lactic acid bacteria: from food to health. Microb Cell Factories..

[14] Azcarate-Peril MA, Altermann E, Hoover-Fitzula RL, Cano RJ, Klaenhammer TR (2004). Identification and inactivation of genetic loci involved with lactobacillus acidophilus acid tolerance. Appl Environ Microbiol..

[15] Bron PA, Marco M, Hoffer SM, Van Mullekom E, de Vos WM, Kleerebezem M (2004). Genetic characterization of the bile salt response in lactobacillus plantarum and analysis of responsive promoters in vitro and in situ in the gastrointestinal tract. J Bacteriol..

[16] Botes M, van Reenen CA, Dicks LMT (2008). Evaluation of enterococcus mundtii ST4SA and lactobacillus plantarum 423 as probiotics using a gastro-intestinal model with infant milk formulations as substrate. Int J Food Microbiol.

[17] Molly K, Van de Woestyne M, Verstraete W (1993). Development of a 5-step multi-chamber reactor as a simulation of the human intestinal microbial ecosystem. Appl Microbiol Biotechnol..

[18] Macfarlane GT, Macfarlane S, Gibson GR (1998). Validation of a three-stage compound continuous culture system for investigating the effect of retention time on the ecology and metabolism of bacteria in the human colon. Microb Ecol..

[19] Mainville I, Arcand Y, Farnworth ER (2005). A dynamic model that simulates the human upper-gastro-intestinal tract for the study of probiotics. Int J Food Microbiol..

[20] Andrue N, Zelmer A, Wiles S (2001). Noninvasive biophotonic imaging for studies of infectious disease. FEMS Microbiol Rev..

[21] Van Zyl WF, Deane SM, Dicks LMT (2015). Reporter systems for in vivo tracking of lactic acid bacteria. Gut Microbes..

[22] Wiles S, Robertson BD, Frankel G, Kerton A (2009). Bioluminescent monitoring of in vivo colonization and clearance dynamics by light-emitting bacteria. Methods Mol Biol..

[23] Foucault ML, Thomas L, Goussard S, Branchini BR, Grillot-Courvalin C (2001). *In vivo* bioluminescence imaging for the study of intestinal colonization by *Escherichia coli* in mice. Appl Environ Microbiol..

[24] Van Zyl WF, Deane SM, Dicks LMT (2015). Use of the mCherry fluorescent protein to study intestinal colonization by *Enterococcus mundtii* ST4SA and *Lactobacillus plantarum* 423 in mice. Appl Environ Microbiol..

[25] Berlec A, Završnik J, Butinar M, Turk B, Štrukelj B (2015). *In vivo* imaging of *Lactococcus lactis*, *Lactobacillus plantarum* and *Escherichia coli* expressing infrared fluorescent protein in mice. Microb Cell Fact..

[26] Oozeer R, Goupil-Feuillerat N, Alpert CA, van de Guchte M, Anba J, Mengaud J (2002). *Lactobacillus casei* is able to survive and initiate protein synthesis during its transit in the digestive tract of human flora-associated mice. Appl Environ Microbiol..

[27] Eom J, Ahn W, Her S, Moon G (2014). Construction of bioluminescent Lactobacillus casei CJNU 0588 for murine whole body imaging. Food Sci Biotechnol..

[28] Szittner R, Meighen E (1990). Nucleotide sequence, expression, and properties of luciferase coded by lux genes from a terrestrial bacterium. J Biol Chem..

[29] Cronin M, Sleator RD, Hill C, Fitzgerald GF, van Sinderin D (2008). Development of luciferase-based reporter system to monitor *Bifidobacterium breve* UCC2003 persistence in mice. BMC Microbiol..

[30] Corthier G, Delorme C, Ehrlich SD, Renault P (1998). Use of luciferase genes as biosensors to study bacterial physiology in the digestive tract. Appl Environ Microbiol..

[31] Lee M, Moon G (2012). *In vivo* imaging of *Escherichia coli* and *Lactococcus lactis* in murine intestines using a reporter luciferase gene. Food Sci Biotechnol..

[32] La Rosa SL, Casey PG, Hill C, Diep DB, Nes IF, Brede DA (2013). In vivo assessment of growth and virulence gene expression during commensal and pathogenic lifestyles of luxABCDE-tagged enterococcus faecalis strains in murine gastrointestinal and intravenous infection models. Appl Environ Microbiol..

[33] Daniel C, Poiret S, Dennin V, Boutillier D, Pot B (2013). Bioluminescence imaging study of spatial and temporal persistence of *Lactobacillus plantarum* and *Lactococcus lactis* in living mice. Appl Environ Microbiol..

[34] Van Reenen CA, Dicks LMT, Chikindas ML (1998). Isolation, purification and partial characterization of plantaricin 423, a bacteriocin produced by *Lactobacillus plantarum*. J Appl Microbiol..

[35] Knoetze H, Todorov SD, Van Reenen CA, Dicks LMT. Characterization of bacteriocin ST4SA, produced by Enterococcus mundtii ST4SA isolated from soya beans. Department of Microbiology, Stellenbosch University, South Africa, S.A. 2006.

[36] Ramiah K, ten Doeschate K, Smith R, Dicks LMT (2009). Safety assessment of *Enterococcus mundtii* ST4SA and *Lactobacillus plantarum* 423 determined in trials with Wistar rats. Probiot Antimicrob Prot..

[37] Botes M, Loos B, van Reenen CA, Dicks LMT (2008). Adhesion of the probiotic strains *Enterococcus mundtii* ST4SA and *Lactobacillus plantarum* 423 to Caco-2 cells under conditions simulating the intestinal tract, and in the presence of antibiotics and inflammatory medicaments. Arch Microbiol..

[38] Mare L, Wolfaardt GM, Dicks LMT (2006). Adhesion of *Lactobacillus plantarum* 423 and *Lactobacillus salivarus* 241 to the intestinal tract of piglets, as recorded with fluorescent *in situ* hybridisation (FISH) and production of plantaricin 423 by cells colonized to the ileum. J Appl Microbiol..

[39] Granger M, van Reenen CA, Dicks LMT (2007). Effect of gastrointestinal conditions on the growth of *Enterococcus mundtii* ST4SA, and production of bacteriocin ST4SA recorded by real-time PCR. Int J Food Microbiol..

[40] Ramiah K, van Reenen CA, Dicks LMT (2008). Surface-bound proteins of *Lactobacillus plantarum* 423 that contribute to adhesion of Caco-2 cells and their role in competitive exclusion and displacement of *Clostridium sporogenes* and *Enterococcus faecalis*. Res Microbiol..

[41] Dicks LMT, ten Doeschate K (2010). *Enterococcus mundtii* ST4SA and *Lactobacillus plantarum* 423 alleviated symptoms of *Salmonella* infection, as determined in Wistar rats challenged with *Salmonella enterica* serovar typhimurium. Curr Microbiol..

[42] Van Zyl WF, Deane SM, Dicks LMT (2015). *Enterococcus mundtii* ST4SA and *Lactobacillus plantarum* 423 excludes *Listeria monocytogenes* from the GIT, as shown by bioluminescent studies in mice. Benefic Microbes..

[43] Hugenholtz F, de Vos WM (2018). Mouse models for human intestinal microbiota research: a critical evaluation. Cell Mol Life Sci..

[44] Mierau I, Kleerebezem M (2006). 10 years of the nisin-controlled gene expression system (NICE) in *Lactococcus lactis*. Appl Microbiol Biotechnol..

[45] Thomas CM (2000). Paradigms of plasmid organization. Mol Microbiol..

[46] Tauer C, Heinl S, Egger E, Heiss S, Grabher R (2014). Tuning constitutive recombinant gene expression in *Lactobacillus plantarum*. Microb Cell Factories..

[47] Sasikumar P, Gomathi S, Anbazhagan K, Baby AE, Sangeetha J, Selvam GS (2014). Genetically engineered *Lactobacillus plantarum* WCFS1 constitutively secreting heterologous oxalate decarboxylase and degrading oxalate under *in vitro*. Curr Microbiol..

[48] Karimi S, Ahl D, Vågesjö E, Holm L, Philipson M, Jonsson H (2016). *In vivo* and *in vitro* detection of luminescent and fluorescent *Lactobacillus reuteri* and application of red fluorescent mCherry for assessing plasmid persistence. PLoS One..

[49] Alander M, De Smet I, Nollet L, Verstraete W, von Wright A, Mattila-Sandholm T (1999). The effect of probiotic strains on the microbiota of the simulator of the human intestinal microbial ecosystem (SHIME). Int J Food Microbiol.

[50] Su P, Henriksson A, Tandianus JE, Park JH, Foong F, Dunn NW (2005). Detection and quantification of *Bifidobacterium lactis* LAFTI B94 in human faecal samples from a consumption trial. FEMS Microbiol Lett..

[51] Johansson ML, Nobaek S, Berggren A, Nyman M, Bjorck I, Ahrne S (1999). Survival of *Lactobacillus plantarum* DSM 9843 (299v), and effect on the short-chain fatty acid content of faeces after ingestion of a rose-hip drink with fermented oats. Int J Food Microbiol..

[52] Fujiwara S, Seto Y, Kimura A, Hashiba H (2001). Establishment of orally-administered lactobacillus gasseri SBT2055SR in the gastrointestinal tract of humans and its influence on intestinal microflora and metabolism. J Appl Microbiol..

[53] Dean-Nystrom EA, Bosworth BT, Moon HW (1999). Pathogenesis of *Escherichia coli* O157:H7 in weaned calves. Adv Exp Med Biol..

[54] Wiles S, Clare S, Harker J, Huett A, Young D, Dougan G (2004). Organ specificity, colonization and clearance dynamics *in vivo* following oral challenges with the murine pathogen *Citrobacter rodentium*. Cell Microbiol..

[55] Trcek J, Fuchs TM, Trulzsch K (2010). Analysis of *Yersinia enterocolitica* invasin expression *in vitro* and *in vivo* using a novel luxCDABE reporter system. Microbiology..

[56] Baumler AJ, Tsolis RM, Heffron F (1996). The lpf fimbrial operon mediates adhesion of *Salmonella typhimurium* to murine Peyer's patches. Proc Natl Acad Sci U S A..

[57] Andrue N, Zelmer A, Sampson SL, Ikeh M, Bancroft GJ, Schaible UE (2013). Rapid *in vivo* assessment of drug efficacy against *Mycobacterium tuberculosis* using an improved firefly luciferase. J Antimicrob Chemother..

[58] Rhee KJ, Cheng H, Harris A, Morin C, Kaper JB, Hecht G (2011). Determination of spatial and temporal colonization of enteropathogenic *E. coli* and enterohemorrhagic *E. coli* in mice. Gut Microbes..

