# Supplementary material for: In vivo bioluminescence imaging of the spatial and temporal colonization of lactobacillus plantarum 423 and enterococcus mundtii ST4SA in the intestinal tract of mice
Source: BMC Microbiol. 2018 Oct 30;18:171. doi: 10.1186/s12866-018-1315-4 (PMC6208077; doi:10.1186/s12866-018-1315-4)
Supplement: Supplementary file 1 — Figure S1. In vitro stability of bioluminescence. (a) Stability of plasmid pNZPldhFfluc in L. plantarum 423 Fluc and plasmid pNZSTldhFfluc in E. mundtii ST4SA Fluc after subculturing for 7 days with replica plating on non-selective (antibiotic-free) and selective (Cm) media. The percentages of Cm-resistant colonies of three independent cultures of each respective strain are shown. Bioluminescent colonies of (b) L. plantarum 423 Fluc and (c) E. mundtii ST4SA Fluc after 7 days of subculture in antibiotic-free MRS media. (PDF 515 kb) [file 12866_2018_1315_MOESM1_ESM.pdf]

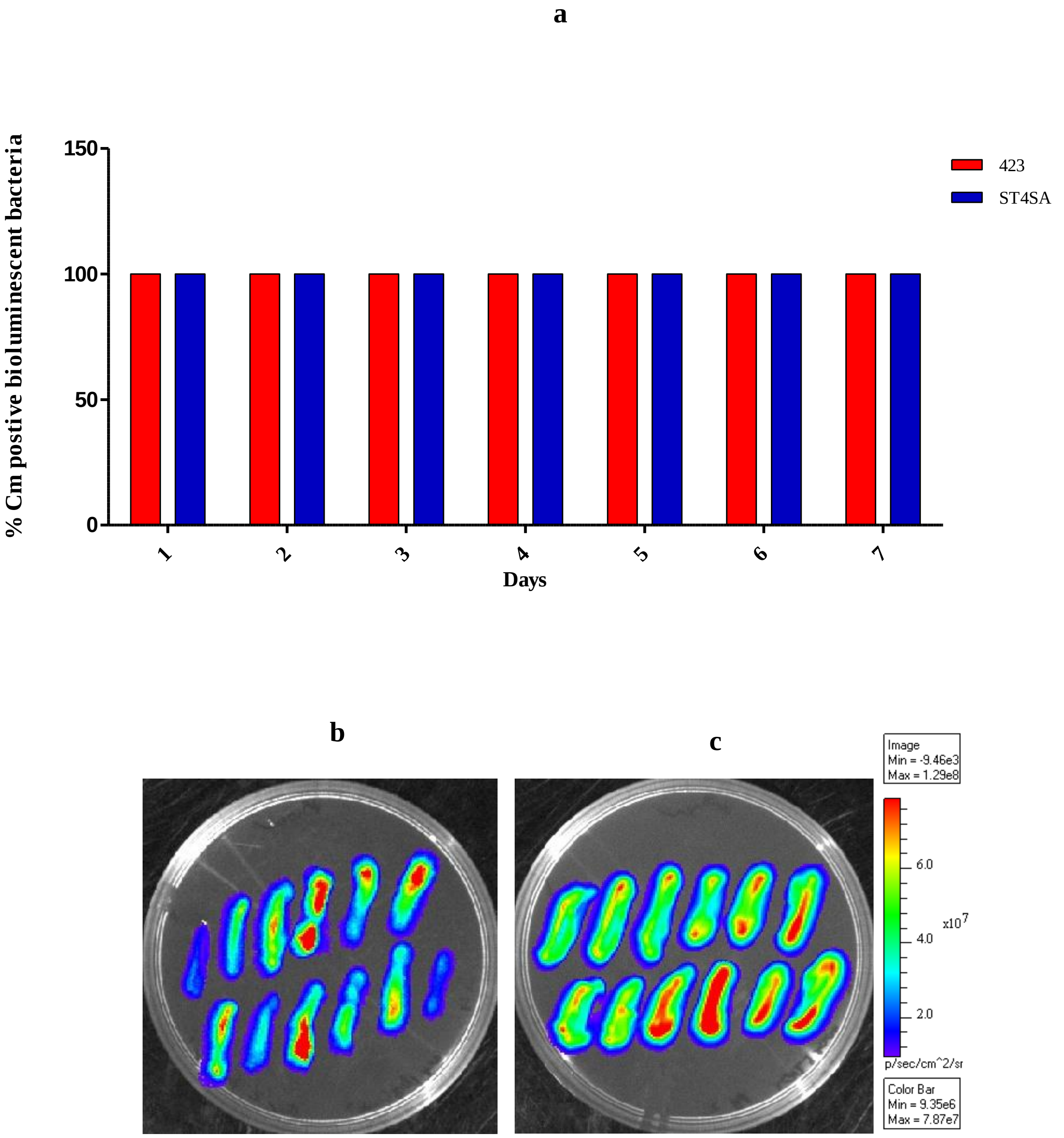

Figure S1. *In vitro* stability of bioluminescence. **(a)** Stability of plasmid pNZPldhFfluc in *L. plantarum* 423 Fluc and plasmid pNZSTldhFfluc in *E. mundtii* ST4SA Fluc after subculturing for 7 days with replica plating on non-selective (antibiotic-free) and selective (Cm) media. The percentages of Cm-resistant colonies of three independent cultures of each respective strain are shown. Bioluminescent colonies of **(b)** *L. plantarum* 423 Fluc and **(c)** *E. mundtii* ST4SA Fluc after 7 days of subculture in antibiotic-free MRS media.
