# Supplementary material for: In vivo bioluminescence imaging of the spatial and temporal colonization of lactobacillus plantarum 423 and enterococcus mundtii ST4SA in the intestinal tract of mice
Source: BMC Microbiol. 2018 Oct 30;18:171. doi: 10.1186/s12866-018-1315-4 (PMC6208077; doi:10.1186/s12866-018-1315-4)
Supplement: Supplementary file 2 — Table S1. Primers used in this study. (PDF 497 kb) [file 12866_2018_1315_MOESM2_ESM.pdf]

Table S1. Primers used in this study

Target	**P**rimer	Sequence (5' to 3')†	Restriction sites	Product size (bp)
P*ldh*	Pldh1	GCGC**AGATCT**AATCTTCTCACCGTCTTG	*Bgl*II	520
	Pldh2	ATAT**CCATGG**TAAGTCATCCTCTCGTAGTG	*Nco*I	
ST*ldh*	ldhS1	GCGC**AGATCT**GAAGAACACAATCCGACT	*Bgl*II	166
	ldhS2	TCCT**CCATGG**TCTAACACACCTTTCA	*Nco*I	
ffluc	FlucFor	ATAT**CCATGG**AGGACGCTAAGAACATCA	*Nco*I	1669
	FlucRev	GCGC**TCTAGA**CACAATTTCGACTTGCCAC	*Xba*I	

†: Bold and underlined sequences indicate restriction sites. Full target names are listed in main text.
