# Supplementary material for: In vivo bioluminescence imaging of the spatial and temporal colonization of lactobacillus plantarum 423 and enterococcus mundtii ST4SA in the intestinal tract of mice
Source: BMC Microbiol. 2018 Oct 30;18:171. doi: 10.1186/s12866-018-1315-4 (PMC6208077; doi:10.1186/s12866-018-1315-4)
Supplement: Supplementary file 3 — Figure S2. Schematic representing the construction of the pNZPldhFfluc and pNZSTldhFfluc luciferase expression plasmids. Relevant features are indicated, including restriction sites and PCR primers used for cloning; the E. coli/LAB repA and repC replication genes; the chloramphenicol acetyltransferase (cat) gene conferring resistance to chloramphenicol; the Pldh promoter from the L. plantarum 423 lactate dehydrogenase gene and the STldh promoter from the E. mundtii ST4SA lactate dehydrogenase gene. (PDF 507 kb) [file 12866_2018_1315_MOESM3_ESM.pdf]

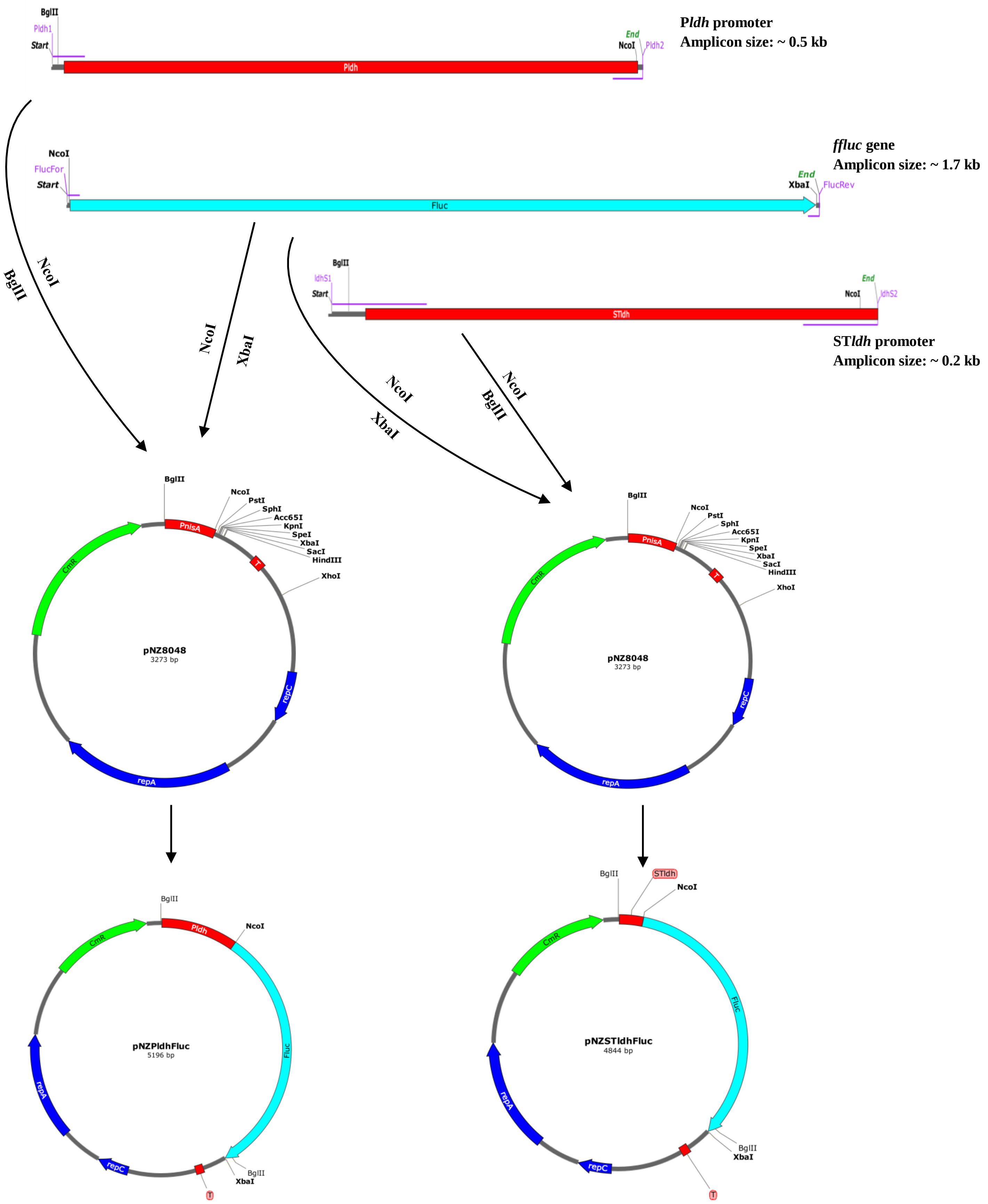
BglII
Pldh1
Start
Pldh
End
NcoI
Pldh2
P*ldh* promoter
Amplicon size: ~ 0.5 kb
NcoI
FlucFor
Start
Fluc
End
XbaI
FlucRev
ffluc gene
Amplicon size: ~ 1.7 kb
BglII
ldhS1
Start
STldh
End
NcoI
ldhS2
ST*ldh* promoter
Amplicon size: ~ 0.2 kb
BglII
NcoI
NcoI
XbaI
NcoI
XbaI
NcoI
BglII
BglII
NcoI
PstI
SphI
Acc65I
KpnI
SpeI
XbaI
SacI
HindIII
XhoI
PnisA
T
CmR
repC
repA
pNZ8048
3273 bp
BglII
NcoI
PstI
SphI
Acc65I
KpnI
SpeI
XbaI
SacI
HindIII
XhoI
PnisA
T
CmR
repC
repA
pNZ8048
3273 bp
BglII
NcoI
Pldh
CmR
Fluc
repA
repC
T
BglII
XbaI
pNZPldhFluc
5196 bp
BglII
STldh
NcoI
CmR
Fluc
repA
repC
T
BglII
XbaI
pNZSTldhFluc
4844 bp

Figure S2. Schematic representing the construction of the pNZPldhFfluc and pNZSTldhFfluc luciferase expression plasmids. Relevant features are indicated, including restriction sites and PCR primers used for cloning; the *E. coli*/LAB *repA* and *repC* replication genes; the chloramphenicol acetyltransferase (*cat*) gene conferring resistance to chloramphenicol; the P*ldh* promoter from the *L. plantarum* 423 lactate dehydrogenase gene and the ST*ldh* promoter from the *E. mundtii* ST4SA lactate dehydrogenase gene.
